# Hypoxia-inducible factors link inflammation and lipid metabolism in atherosclerotic macrophages

**DOI:** 10.3389/fcvm.2026.1765661

**Published:** 2026-02-09

**Authors:** Kyu Seong Park, Yu Jin Ko, Jae-Hoon Choi

**Affiliations:** Laboratory of Pathophysiology, Department of Life Science, College of Natural Sciences, Research Institute for Natural Sciences, Hanyang Institute of Bioscience and Biotechnology, Hanyang University, Seoul, Republic of Korea

**Keywords:** agonist/antagonist, atherosclerosis, hypoxia-inducible factor, inflammation, lipid metabolism, macrophage

## Abstract

Atherosclerosis is a chronic inflammatory disease driven by a complex interplay between immune cells, inflammation, metabolic dysfunction, and hypoxia. Among immune cells, macrophages interact bidirectionally with these factors, undergoing phenotypic and functional changes in response to the microenvironment, which contribute to both the progression and resolution of atherosclerosis. Recent studies have elucidated these dynamic interactions among these factors; however, research remains focused on individual aspects, and a more integrated understanding has yet to be fully established. Therefore, this review aimed to emphasize the importance of complex interactions among hypoxia, inflammation, and lipid metabolism, suggesting that the crosstalk among these response pathways operates actively and dynamically rather than following a simple cause-and-effect pathway. Further, this review highlights recent advances in understanding the inflammatory functions of hypoxia-inducible factor-1α and hypoxia-inducible factor-2α in macrophages under hypoxic stress. In addition, we explore the systemic implications of HIF signaling in lipid-regulating organs such as the liver, intestine, and adipose tissue, emphasizing the emerging paradigm that HIF-2α acts as a metabolic switch coordinating lipid accumulation and inflammation. Finally, we summarize therapeutic approaches targeting HIFs, including HIF stabilizers and HIF-2α-selective antagonists. Collectively, this review offers a comprehensive multi-organ perspective on the immune-metabolic roles of HIFs in atherosclerosis, providing valuable insights into future therapeutic interventions.

## 1 Introduction

Atherosclerosis is a chronic inflammatory disease and a major cause of acute heart failure, myocardial infarction (MI), and stroke, contributing to high global mortality (1). Since atherosclerosis was initially defined in the late 20th century, extensive research has been conducted continuously on prevention and treatment. Advanced methods in basic scientific research and medical technology have significantly improved our understanding of atherosclerosis (2). Recently, patients have benefited from managing atherosclerosis through stent implantation procedures and statin-based pharmacological interventions. However, these treatments do not provide a fundamental solution or prevention. Therefore, further research on the pathogenesis of atherosclerosis is necessary to inhibit its progression and promote its regression.

Atherosclerosis is initiated by the accumulation of oxidized low-density lipoproteins (oxLDLs) in the aortic intimal layer, which induces inflammation (3). The inflammatory response increases cellular oxygen consumption, which may contribute to ischemic hypoxia as the oxygen supply does not increase proportionally (4, 5). Given that atherosclerosis is a chronic disease, oxygen tension likely undergoes gradual and dynamic changes that influence not only the plaque itself but also the surrounding aortic region. It has been well-established that hypoxia-inducible factors (HIF-1α and HIF-2α) become stabilized under hypoxic conditions (6). Stabilization or degradation of HIFs is regulated by prolyl hydroxylases (PHDs), which are activated in the cytoplasm under efficient oxygen levels. Activated PHDs hydroxylate HIFs, inducing von Hippel–Lindau (VHL) binding and subsequent ubiquitination and proteasomal degradation of HIFs (7). HIFs must bind with HIF-1β/ARNT to form a heterodimeric complex, which translocate into the nucleus. This dimer can bind to specific genes that contain a hypoxia response element (HRE) region. Whereas the HIF-1α/ARNT complex binds to promoter regions within 50 kb, the HIF-2α/ARNT complex prefers to bind to enhancer regions within 5–500 kb (8, 9). In addition, the same genes may be regulated by either HIF-1α or HIF-2α, depending on cell type (10). This overlap suggests that HIF isoforms cannot be considered in strict isolation; rather their contributions depends on cell context, stimulus, and duration of hypoxia. For instance, the expression of HIF-1α is upregulated in inflammation-induced hypoxia within tumors (11). HIF-1α levels increase rapidly in response to hypoxia, exacerbating inflammation and promoting alternative metabolic pathways, such as glycolysis, and over oxygen-consuming processes, including the citric acid cycle (12). Conversely, HIF-2α remains stable throughout the early stages of hypoxia and is sustained for a longer period, playing an anti-inflammatory role (13, 14). Studies have confirmed the presence of hypoxia in human and murine atherosclerotic lesions (4, 7). Since hypoxia-driven microenvironmental changes can alter the behavior of various cell types within atherosclerotic lesions, it is important to investigate the effects of hypoxic conditions on the progression of atherosclerosis. Several studies have reported that HIFs are activated in various tissues and cell types under low oxygen level. These studies suggest that HIF-1α in endothelial cells is involved in angiogenesis and vascular remodeling, whereas HIF-2α regulates lipid metabolism and systemic inflammation in hepatocytes and adipocytes (15–17). These diverse cellular contexts indicate that HIF signaling integrates multiple tissue responses, thereby influencing the overall progression of atherosclerosis.

Although hypoxia affects multiple cell types within arteries, it has a particularly significant influence on macrophages, which are activated by inflammation in atherosclerotic lesions. Meta-analyses of single-cell RNA sequencing (scRNA-seq) datasets identified diverse macrophage subtypes with distinct roles in the atherosclerotic aorta (18). Macrophage subsets in atherosclerosis can be categorized into three groups based on their positions or functions: adventitial macrophages, intimal non-foamy macrophages, and intimal foamy macrophages. Resident adventitial macrophages are responsible for maintaining homeostasis, while non-foamy macrophages are proinflammatory and express inflammatory cytokines. Among known macrophage subtypes, foamy macrophages express both anti-inflammatory genes and lipid metabolism-related genes to clear accumulated lipoproteins in the cells (19). Researchers have attempted to analyze the lipidome of macrophages in the atherosclerotic aorta and integrate these data with sequencing results to determine whether foamy macrophages can eliminate oxLDLs and aid in resolving atherosclerosis (20, 21). Such multi-omics analyses are important for understanding the functions of foamy macrophages and their potential role in mitigating atherosclerosis.

Traditional *in vitro* studies have several limitations as they focus on characterizing specific cell types without considering the microenvironment and interactions with other cell types in the tissue. To address these limitations, spatial transcriptomics have emerged as a powerful technique to visualize the transcriptome within various cell types in tissues. Additionally, serial tissue sections can be stained with multiple antibodies to confirm protein-level results, facilitating the direct visualization of cell-to-cell interactions and integrating transcriptomic and spatial analysis data. These approaches enhance the understanding of atherosclerosis and provide opportunities to develop effective strategies for its treatment and prevention.

This review summarizes the interconnections between key topics in atherosclerosis research, including hypoxia, macrophages, and their lipidome, by integrating recent findings with cutting-edge technologies to provide insights that may guide future therapeutic and preventive strategies for atherosclerosis.

## 2 Macrophage heterogeneity in atherosclerosis

Macrophages are representative of innate immune cells that can eliminate pathogens directly through phagocytosis and secrete inflammatory cytokines to recruit other immune cells and present the antigens to lymphocytes. Interestingly, macrophages also suppress inflammation to prevent tissue damage from prolonged inflammatory responses. Researchers have focused on the plasticity of macrophage phenotypes, which play an important role in understanding the progression and resolution of inflammation in diseased areas (1, 22). Atherosclerosis is initiated by the accumulation of oxLDL in the aortic intima, which induces inflammation and the infiltration of circulating monocytes into the intima. Monocytes are differentiated into macrophages in the inner layer; then, these naïve macrophages are activated by oxLDL to trigger inflammatory responses. Although activated macrophages also digest oxLDL to clean up the aorta, excessive oxLDL uptake by the phagocytic macrophages makes them infeasible for digestion, ultimately becoming foamy macrophages. Efferocytosis by newly differentiated macrophages is enhanced to clear damage-associated molecular patterns (DAMPs) derived from apoptotic or necroptotic foam cells in the intima layer. These serial sequences contribute to the development of larger plaques and a necrotic core in the lesion area. Macrophages in the plaque differentiate into diverse subtypes with distinct gene expressions due to the complex microenvironment, playing unique roles in enhancing or resolving inflammation.

Recent studies using scRNA-seq data have confirmed the existence of diverse subsets of macrophages in the atherosclerotic aortas of high-fat diet-induced Ldlr knockout (KO) mice (21, 23). Four macrophage subtypes were identified in a meta-analysis of nine datasets from CyTOF and scRNA-seq: resident macrophages, inflammatory macrophages, Trem2^+^ foamy macrophages, and IFN-inducible cells (IFNICs), which shared *Cd11b/Itgam*, *Cd64/Fcgr1*, and *Cd68* expression, representative macrophage markers (24). Resident macrophages derived from the yolk sac mainly express *Cd206, Lyve1*, and *Cx3cr1*, which are involved in protecting against antigens and maintaining homeostasis (21, 23). Although these homeostatic macrophages are primarily located in the vascular adventitia, previous research suggests that resident macrophages also exist in the intima as aortic intimal resident macrophages (Mac^AIR^) (25). Inflammatory macrophages are recognized as the primary inducers of atherosclerosis, expressing inflammatory cytokines that recruit immune cells into the intima and contribute to atheroma development (23, 26). Since Trem2^+^ foamy macrophages absorb accumulated oxLDL in the intima through CD36 or scavenger receptor A (SR-A), these cells appear lipid-laden (27, 28). Comprehensive transcriptomic analyses suggest that Trem2^+^ foamy macrophages perform an athero-protective role, rather than the initial determination of being inflammatory macrophages that exacerbate atherosclerosis (21). This paradigm shift is supported by the observation that foamy macrophages exhibit anti-inflammatory properties and contribute to resolving plaque inflammation in a Trem2-dependent manner (29, 30). IFNICs are a minor subset whose roles in atherosclerosis have yet to be investigated; however, it is well established that IFNICs express type I interferons, such as *Isg15*, *Oasl2, and Irf9* (18, 21).

## 3 Hypoxia and macrophages in atherosclerosis

### 3.1 Hypoxia in atherogenesis

Hypoxia is observed in both human and animal models. Pimonidazole hydrochloride, a chemical probe for tissue hypoxia, detected hypoxic regions in human atherosclerotic carotid arteries, yet not in non-diseased aorta (4, 31). The low oxygen level in atherosclerotic plaques exacerbates vascular impairments, as hypoxia-induced angiogenesis from the vasa vasorum supplies oxygen to the plaque (32). Newly developed vessels in the plaque cause destabilization, resulting in enhanced thrombosis and the infiltration of additional immune cells (33, 34). Furthermore, hypoxia stimulates the activation of the inflammasome in macrophages, increasing the secretion of inflammatory cytokines (35). Another report suggested that hypoxic conditions suppress the migration of macrophages in the lesion area, promoting the accumulation of innate immune cells and lipids by disrupting ABCA1 (7, 36). Thus, plaque hypoxia is generally associated with necrotic core expansion due to immune cell exhaustion, reducing efferocytosis (31).

**Figure 1:**
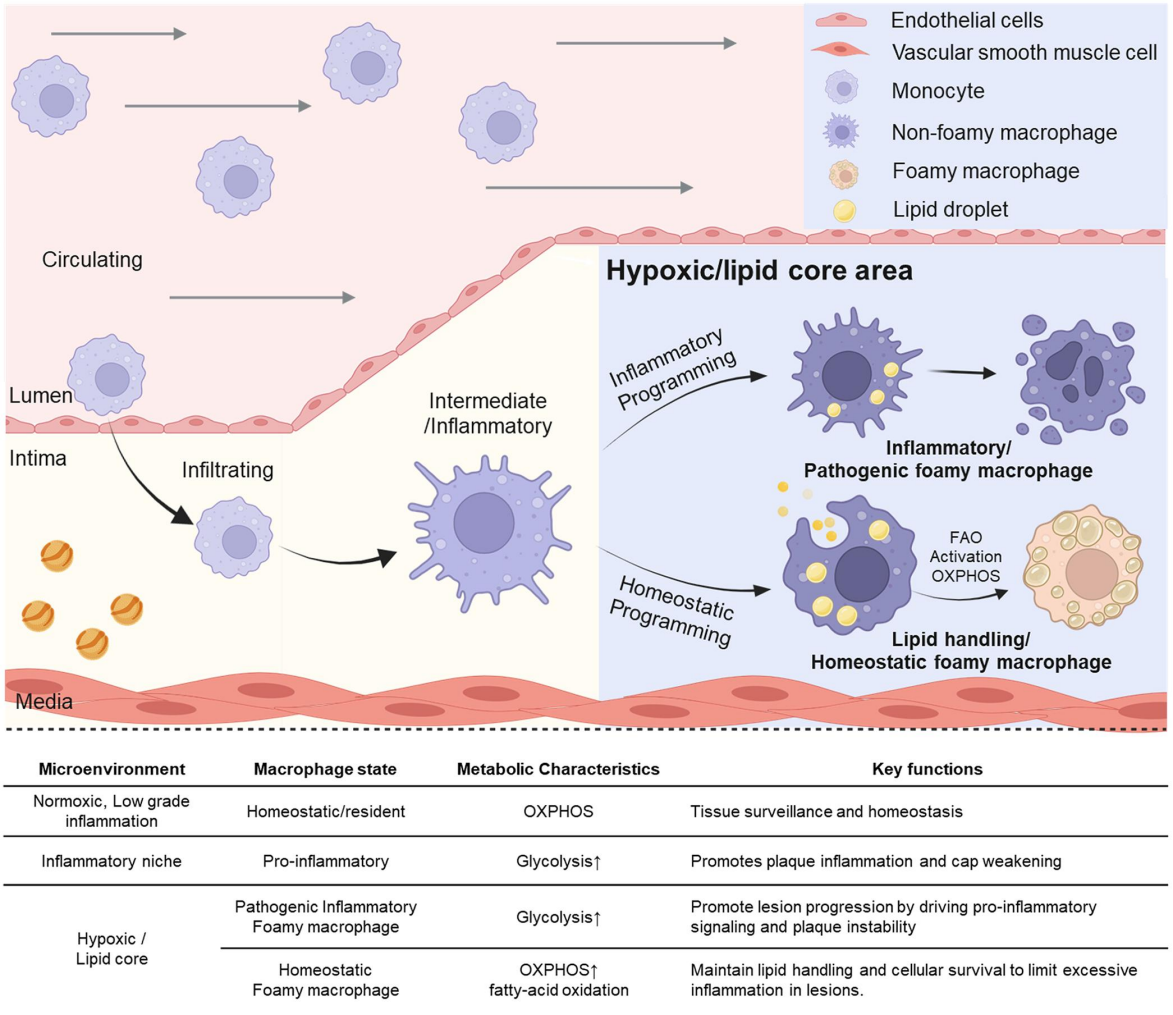
Transition of macrophage subtypes and their distinct function due to changes of microenvironment in process of atherogenesis. Circulating monocytes infiltrate the intimal layer and differentiate into macrophages. These macrophages undergo inflammatory or homeostatic programming depending on microenvironmental cues such as hypoxic lesions or lipid-rich core regions. While the inflammatory niche promotes pro-inflammatory signaling through pathogenic macrophages, homeostatic programming gives rise to lipid-handling foamy macrophages that activate fatty acid oxidation (FAO) and oxidative phosphorylation (OXPHOS), thereby supporting tissue homeostasis. The upper right panel shows schematic icons representing endothelial cells, vascular smooth muscle cells, monocytes, macrophages, foam cells, and lipid droplets. Created in BioRender. Choi, J. (2026) https://BioRender.com/g7ryedj, licensed under Academic License.

Mechanistically, HIFs orchestrate these dynamic alterations in the lesion microenvironment in response to hypoxia. Figure 1 illustrates the transitions of macrophage subtypes and their major functions in response to changes in the atherogenic microenvironment. Since HIFs are stabilized under low-oxygen conditions, hypoxia in atherosclerotic lesions stabilizes the HIFs in the human and mouse aortas (4, 7). Among many attempts to identify the influence of PHDs on the progression of atherosclerosis (37–41), Van Kuijk et al. utilized three mouse models deficient in PHD1, PHD2, and PHD3 (40). Among these, HIF-1α stabilization was observed in myeloid PHD2-deficient mice, resulting in a remarkable phenotype. According to the results of transcriptomic analysis, the expression of *Bnip3* mRNA, which is related with apoptosis, was increased, while collagen content and fibrous cap thickness were significantly elevated (40). These findings suggest that myeloid PHD2 deficiency exacerbates atherosclerosis by stabilizing HIF-1α and promoting plaque fibrosis through macrophage–fibroblast interactions (40). Another experiment reported that an overactive inflammatory response in myeloid cells, particularly macrophages, was induced after bone marrow transplantation or macrophage depletion (41). Since NF-κB and HIF-1α were activated in PHD3-deficient macrophages, M1 polarization and cytokine secretion were increased *in vitro* (41). These studies suggest that HIFs influence the differentiation of macrophages into M1-like or M2-like phenotypes via biological pathways, and HIFs control the main functions of macrophages, including apoptosis and inflammation. Consistent with these findings, however, it is remains controversial that whole-body PHD3 deficiency in Ldlr KO mice leads to increased plasma lipid levels and hematocrit without altering atherosclerotic plaque size, suggesting that chronic HIF-2α stabilization may induce systemic metabolic disturbances without improving vascular outcomes (42).

### 3.2 General roles of HIF-1α and HIF-2α in macrophages

Although both HIF-1α and HIF-2α share structural similarities and activate some genes as transcription factors, they also regulate unique genes in different ways. Macrophages exhibit distinct patterns caused by different inflammatory or metabolic responses depending on whether HIF-1α or HIF-2α is involved. It is essential to understand the difference between HIF-1α and HIF-2α in the pathophysiological aspects of HIF-mediated macrophage polarization. Polarization of macrophages promotes the development of two distinct macrophage subtypes: proinflammatory M1-like and anti-inflammatory M2-like macrophages. These different phenotypes exhibit dynamic plasticity in response to various circumstances or stimuli, including hypoxia, metabolic stress, and inflammation (43). Since HIFs are activated under hypoxic conditions, current research is investigating the role of HIF-1α and HIF-2α in the effects on macrophage phenotypes. HIF-1α is related to the M1-like phenotype, which is mostly activated in inflammatory states. HIF-1α upregulates inflammatory cytokines, including TNF-α and IL-6, as well as NOS2 (iNOS) expression, enhancing nitric oxide (NO) production within the cell to protect against pathogens (14). Furthermore, NO generated by HIF-1α sustains the TCA cycle by activating the aspartate–arginosuccinate shunt for inflammation in macrophages (44). M1 polarization is influenced by inflammatory cytokines induced by succinate dehydrogenase breakdown in the TCA cycle (12). These HIF-1α-mediated inflammatory responses in macrophages play a more significant role in acute inflammation due to the increased iNOS expression-mediated clearance of pathogens (13). In chronic inflammatory conditions, however, the direct gene targets of HIF-1α are not yet fully defined, and its role remains to be clarified beyond its acute pro-inflammatory functions.

Conversely, HIF-2α has often been linked with M2 polarization and tissue resolution (45, 46). In many settings, HIF-2α upregulates cytokines and genes related to lipid metabolism and wound healing such as *Arg1* (47, 48). Meanwhile, *Arg1* is one of representative HIF-2α-dependent genes known as anti-inflammatory marker that associated with lipid metabolism and downregulating pro-inflammatory genes for preventing the exacerbation of atherosclerosis (16, 49). However, HIF-2α is not exclusively anti-inflammatory. Context-dependent evidence shows that HIF-2α can also promote pro-inflammatory responses, for example, by inducing IL-6 in hepatic macrophages (50). Additionally, HIF-2α enhances macrophage infiltration and survival via regulating CSF1R expression that consists chronic inflammation (51, 52). Such findings demonstrate that HIF-2α functions extend beyond the M2 paradigm, with roles in both inflammatory amplification and tissue repair depending on microenvironmental cues.

In hypoxic conditions, the interaction between HIFs affects macrophages by shifting their phenotypic characteristics. This hypoxia-driven mechanism provides important insights into the role of HIFs in diseases such as cancer, chronic inflammation, and ischemic injury (53). Thus, both HIF-1α and HIF-2α act along a continuum: HIF-1α preferentially drives acute inflammatory responses, while HIF-2α more often supports resolution, but both isoforms can contribute to control the inflammation in distinct ways. HIF-2α promotes cell homeostasis by suppressing *Marco* expression, thereby regulating oxidative stress and efferocytosis in stable macrophages (54). This suggests that HIF-2α regulates the excessive activity of macrophages under normal conditions. Comparatively, HIF-1α can be activated by LPS even under normoxic conditions, strongly enhancing the expression of inflammation-related genes (55). This implies that HIF-1α is crucial in mediating immune responses in response to infected or inflamed macrophages.

**Figure 2:**
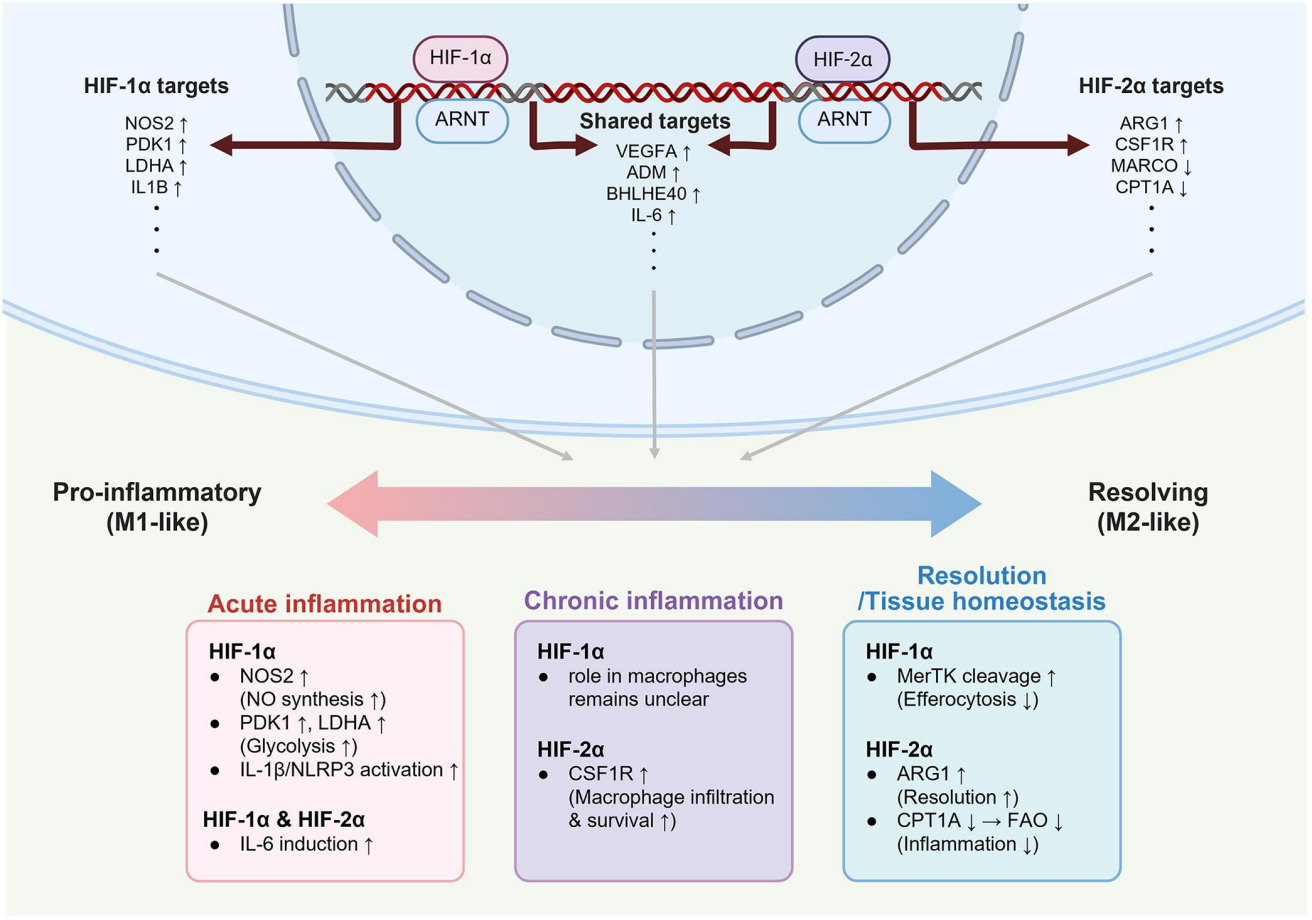
Inflammation and homeostasis mediated by HIF-1α and HIF-2α in different macrophage states. While HIF-1α and HIF-2α share target genes such as *Vegfa*, *Adm*, and *Il6* depending on contextual cues, they also regulate the distinct set of genes, resulting in divergent roles. HIF-1α is preferentially associated with acute inflammation by not only promoting NO synthesis and glycolysis through enhancing *Nos2*, *Pdk1*, and *Ldha*, but also activating the IL-1β/NLRP3 inflammasome. In contrast, HIF-2α up-regulates *Arg1* and *Csf1r* to promote the resolution of chronic inflammation, and down-regulates *Marco* and *Cpt1a* to suppress oxidative stress and inflammatory responses. This schematic illustration is based on findings from murine macrophage studies investigating HIF-dependent metabolic and inflammatory regulation. Created in BioRender. Choi, J. (2026) https://BioRender.com/wrqen81, licensed under Academic License.

Overall, HIF-1α and HIF-2α function through specific pathways to mediate macrophage polarization and regulate macrophage functions. Figure 2 highlights these context-specific and dynamic roles: HIF-1α largely associated with acute inflammatory programs, HIF-2α often linked to resolution, but also capable of contributing to inflammation depending on environmental conditions. Understanding these antagonistic interactions will improve therapeutic strategies targeting HIF-1α and HIF-2α.

### 3.3 Possible roles of HIFs in macrophages for atherosclerosis

HIFs in macrophages have attracted considerable attention in various diseases; HIF-1α is related to M1 polarization, whereas HIF-2α is associated with M2 polarization (14, 56). Inflammatory responses are reduced in HIF-1α-depleted macrophages in atherosclerosis, and the plaque and necrotic core areas are diminished in HIF-1α-deficient mice (40, 57). Previous research using mice suggested that HIF-2α regulates the expression of mitochondrial *Cpt1a* in macrophages, which inhibits the formation of NLRP3 inflammasome complex to suppress inflammation (58). Since inflammation is a key risk factor for atherosclerosis, it could be assumed that HIF-2α-mediated suppression of inflammation regulates the development of atherosclerosis. However, no clear evidence exists that macrophage HIF-2α reduces atherosclerosis. Therefore, further studies on the regulatory mechanisms through which HIF-2α signaling functions are required to understand the development of atherosclerosis.

## 4 Immunometabolism under hypoxia

### 4.1 Crosstalk between inflammation and lipid metabolism

An insufficient oxygen supply to tissue leads to cellular stress, which inhibits physiological processes, including the TCA cycle (59). Subsequently, the cells attempt to reprogram their metabolic processes to generate energy and combat the harsh microenvironment. Immune cells, such as macrophages, require more energy for inflammatory responses. Recently, the immune system–metabolism network has been widely studied to identify the interactions between inflammation and metabolism in cells and tissues (60–62). Inflammatory conditions such as tumors and atherosclerosis are associated with metabolic shifts influencing cellular pathology (63, 64). For instance, foamy macrophages in atherosclerotic lesions exhibit enhanced expression of lipid metabolism–related genes and display anti-inflammatory characteristics (21). Recent findings suggest that the pathological milieu of atherosclerotic plaques not only drives foam cell formation but also modulates their inflammatory output. Consequently, researchers are investigating the regulation of inflammation through foam cell-mediated modulation of lipid metabolism (18, 29, 65). Importantly, inflammation in the atherosclerotic plaques is known to be closely associated with hypoxic conditions (66). While HIFs are key transcriptional regulators, the functional role of these transcription factors in lipid metabolism remains controversial. Thus, it is essential to investigate the emerging paradigm in which foam cells, influenced by the dual stressors of hypoxia and lipid overload, play a role in modulating inflammation within lesions. Ultimately, understanding the roles of HIFs in lesion progression or resolution may offer valuable therapeutic insights into atherosclerosis.

### 4.2 HIFs as drivers of macrophage metabolic programming

Recent comprehensive data revealed that foamy macrophages, which excessively uptake oxLDL, exhibited significantly elevated mRNA expressions of *Fabp4*, *Lgals3*, *Trem2*, *Plin2*, *Lpl*, *Cd36*, and *Abca1* (21, 67). These genes are enriched in the PPARγ signaling pathway, which regulate lipid metabolism. Previous research suggests that PPARγ promotes foam cell formation by regulating the expression of *Fabp4* and *Cd36* in macrophages (28, 68).

HIFs regulate pivotal physiological processes in macrophages under hypoxic conditions. Since HIFs are activated in plaque macrophages in the atheroma of human and mice, several studies have demonstrated the effect of hypoxia on lipid accumulation in macrophages (7, 69). Notably, HIF-1α can regulate lipid metabolism in macrophages by controlling the expression of genes related to lipid metabolism, such as *Lpin1* and *Abca1*, thereby increasing lipid accumulation and exacerbate atherosclerosis (70). Nevertheless, HIF-1α primarily promotes glycolysis in hypoxic conditions by upregulating *Glut1*, *Hk2*, and *Ldha*, which contribute to inflammatory processes and protect against the infections of anaerobic organisms (71, 72).

Lipid uptake by macrophages is increased under hypoxic conditions compared to normoxic conditions; similar results were obtained following oxLDL treatment (7, 73). Thus, HIFs may promote lipid accumulation in macrophages under hypoxic conditions. Notably, the bone marrow-derived macrophages from *Hif2a*-deficient mice exhibited a shift in lipid metabolic gene expression compared to wild-type controls (45). The expressions of genes involved in fatty acid synthesis (*Acaca*, *Fasn*, and *Scd1*) and lipid storage (*Dgat1* and *Plin2*) were markedly reduced. Conversely, genes associated with β-oxidation (*Acox2* and *Cpt1a*) were significantly upregulated. These data indicate that HIF-2α promotes fatty acid synthesis and storage in macrophages while suppressing fatty acid oxidation, thereby contributing to the lipid-laden phenotype observed in hypoxic lesions.

### 4.3 HIFs in hepatocytes

Lipid accumulation in hepatocytes due to chronic alcohol consumption or genetic mutations, can induce non-alcoholic fatty liver disease (NAFLD) and steatosis, which are commonly associated with tissue hypoxia. In this hypoxic condition, HIF-1α controls glucose metabolism, whereas HIF-2α regulates lipid metabolism (74, 75). Notably, overexpression of HIF-2α levels in hepatocytes exacerbates NAFLD or steatosis by increasing lipid accumulation through enhanced *Cd36* expression; however, HIF-1α does not affect *Cd36* expression under hypoxic conditions (76).

Although HIF-1α is essential for cellular adaptation to hypoxic conditions and maintaining glucose homeostasis in hepatocytes, it can also induce fibrosis in NAFLD livers by HIF-1α through the PTEN/p65 signaling pathway activation (75, 77). However, whether HIF-1α exacerbates or attenuates liver fibrosis through lipid metabolism regulation remains controversial (78). For example, HIF-1α has been shown to enhance MCP-1 expression and exacerbate alcoholic liver steatosis via lipid accumulation (79). On the contrary, other studies suggest that HIF-1α mediates lipid metabolism through the PPAR-α/ANGPTL4 pathway or *Lipin1* expression, which suppresses NAFLD progression (80, 81).

Conversely, somatic mutations in the oxygen-dependent domain (ODD) have been reported to stabilize HIF-2α under normoxic conditions in certain NAFLD patients (82, 83). These patients exhibited increased lipid droplets in hepatocytes, as well as elevated liver weight and body weight, resulting from the gain-of-function mutation. Meanwhile, a HIF-2α mutation in hepatocytes enriches the lipid metabolic pathways, especially in HIF-2α mutant mice fed a high-fat diet; these mice also exhibit increased levels of nuclear HIF-2α and its target perilipin-2 (PLIN2) (84). Furthermore, in intestinal *Hif-2*α KO mice, the hepatic expression of fatty acid transport and lipogenesis-related genes such as *Srebp1c*, *Cidea*, *Cd36*, *Fabp4*, *Sdc1*, and *Plin2* was significantly reduced, whereas the expression of β-oxidation-related genes (*Acox2*, *Acsl1*, and *Acaa1a*) was increased (85)*.* These findings highlight the multifaceted roles of HIF-2α in promoting lipid uptake and storage while concurrently suppressing fatty acid catabolism.

Collectively, these studies highlight HIF-2α as a pivotal transcriptional regulator of lipid metabolic programs in hepatocytes. Indeed, HIF-2α plays a significant role in the development and progression of steatosis and fatty liver disease by enhancing lipid accumulation and impairing oxidative lipid clearance. These data provide compelling evidence that HIF-2α is a direct modulator of lipid homeostasis at both cellular and systemic levels.

### 4.4 HIFs in enterocytes

Most studies on atherosclerosis have traditionally focused on vascular and immune cells; however, recent research has suggested that enterocytes regulate systemic lipid homeostasis and inflammatory responses. Enterocytes exhibit unique metabolic adaptations under chronic low-oxygen conditions, which are closely associated with microbial symbiosis and the maintenance of intestinal function. Intestine is sensitive to oxygen concentrations as a major organ responsible for dietary lipid absorption and ceramide synthesis processes that are precisely controlled by HIF signaling (85). Both HIF-1α and HIF-2α act as the metabolic detectors regulating metabolism in hypoxic enterocytes. In the context, understanding the mechanism of metabolic and inflammatory pathways that are regulated by intestinal HIFs is essential for elucidating how intestinal hypoxia indirectly contributes to the systemic inflammatory milieu of atherosclerosis.

HIF-1α is generally expressed in enterocytes, which promotes glucose uptake and glycolysis by regulating the expression of *Glut1* and other glycolysis enzymes (86, 87). GLUT1 transports glucose into cells to increase glucose absorption, thereby activating the PI3K/Akt/mTOR pathways (88). Furthermore, HIF-1α directly interacts with GLUT1 and glycolytic enzymes, including PFKP, to form a glycolytic complex, which promotes glycolysis without transcriptional regulation, ultimately optimizing energy generation through increased lactic acid production (87). An increased lactic acid level helps regulate pH levels in cells, which stimulates the production of occludin to maintain intestinal barrier function (87).

Interestingly, recent research has reported that HIF-2α expression is increased in the ileum of obese patients, whereas HIF-1α levels in biopsies from obese and non-obese patients did not differ (85). According to that, it has been posited that only HIF-2α is upregulated among the HIF family. Additionally, intestinal HIF-2α overexpression through lentivirus–HIF-2α (LV–HIF-2α) transfection into ApoE KO mice increased *Lgals3* expression in macrophages and genes related to ceramide synthesis, whereas macrophage *Lgals3* expression was reduced in intestine from *Hif-2*α KO mice (89). Conversely, *Hif2a* knockout restricted to the intestinal epithelium resulted in reduced *LGALS3* expression, reinforcing the regulatory role of intestinal HIF-2α in this axis.

These findings indicate that intestinal HIF-2α influences atherosclerotic lesion biology indirectly by modulating lipid metabolites and inflammatory mediators. By regulating LGALS3 and ceramide-related pathways, intestinal HIF-2α appears to contribute to the systemic inflammatory milieu that drives lesion progression. This data further contributes to the emerging role of HIF-2α as a tissue-specific orchestrator of lipid metabolism and inflammation across multiple organ systems.

### 4.5 HIFs in adipocytes

Since adipose tissue is metabolically active tissue with both endocrine and immune functions, obesity, a representative disease of adipose tissue, results in chronic inflammation and metabolic dysfunction. Within this diseased microenvironment, HIF signaling regulates lipid metabolism, insulin resistance, and immune activation as a key regulator.

HIF-2α depletion in adipocytes elevates inflammatory responses and enhances insulin resistance, inducing metabolic dysfunction (17). Moreover, HIF-2α regulates *Acer2* expression in adipocytes, which contributes to ceramide catabolism, lowering blood ceramide levels and reducing atherosclerotic lesions (17). Alternatively, HIF-1α can negatively exacerbate obesity and insulin resistance by increasing adipose inflammation via the upregulation of inflammatory cytokines and chemokines (90, 91). Moreover, insulin resistance is closely related to HIF-1α, which increases PDK1/2/4 expression, promoting glycolysis metabolism and enhancing lactic acid production (90). Furthermore, both insulin resistance and inflammation are reduced in the adipose tissues of adipocyte-specific Hif-1α-deficient mice fed high-fat diet by activating the glucagon-like peptide-1 (GLP-1) pathway (92). Conversely, adipocyte HIF-1α overexpression promotes obesity and decreases metabolism through suppressing cell respiration in brown adipose tissue (BAT) (93). Additionally, HIF-1α regulates VEGF expression, which promotes angiogenesis in obese mice (94).

These findings highlight the dual immune–metabolic roles of adipose tissue in health and disease, wherein resident macrophage subtypes and hypoxia-responsive pathways interact to regulate lipid flux, inflammatory tone, and ultimately, cardiovascular risk.

### 4.6 Interplay among hypoxia, inflammation, and metabolism in atherosclerosis

**Figure 3:**
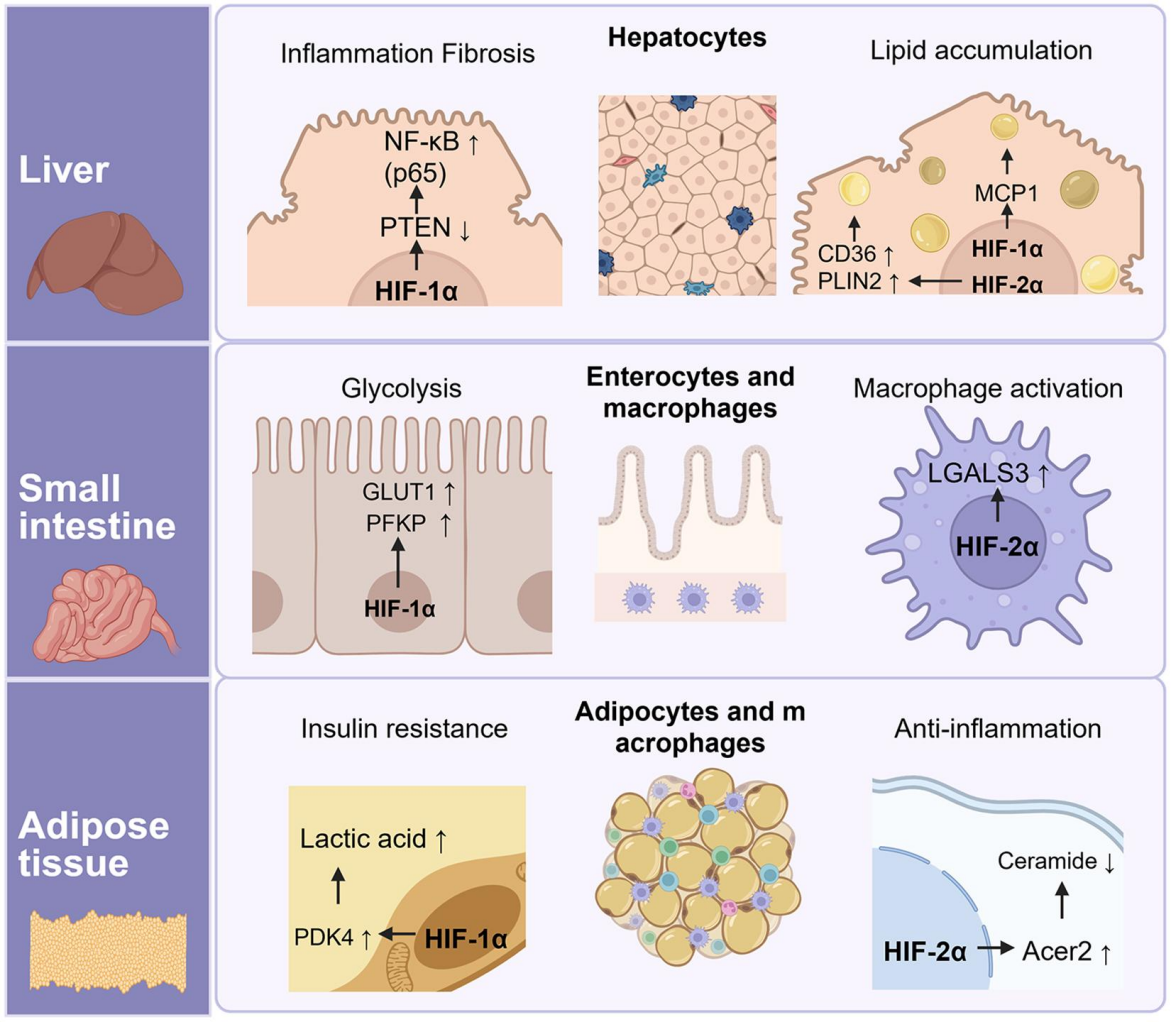
Tissue-specific functions of HIF-1α and HIF-2α in the liver, small intestine, and adipose tissue. HIF-1α downregulates *Pten* expression and consequently activates κB signaling, leading to inflammation and fibrosis in hepatocytes. Lipid accumulation in the liver is mediated by both HIF-1α and HIF-2α: HIF-1α regulates *Mcp1* expression in hepatocytes, whereas HIF-2α controls CD36 and *Plin2* expression in Kupffer cells. In the small intestine, HIF-1α promotes glycolysis by enhancing *Pfkp* and *Glut1* expression, while HIF-2α up-regulates *Lgals3* expression, which contributes to immune responses. Finally, HIF-1α controls PDK1/2/4 expression, leading to lactic acid accumulation and exacerbation of insulin resistance, whereas HIF-2α alleviates ceramide-mediated inflammation via regulating *Acer2* expression in adipose tissue. Created in BioRender. Choi, J. (2026) https://BioRender.com/kgvnjyi, licensed under Academic License.

The interaction between inflammation and metabolism is a complex but important physiological process. Figure 3 summarizes an overview of HIF activity across major organs involved in physiological processes. In macrophages, inflammation either regulates metabolism or is regulated by the byproducts of metabolism (61, 95). The mechanisms involved in immunometabolism are complex but essential for maintaining cellular homeostasis. The immunometabolic pathways are activated under stressful conditions, such as in tumor microenvironments, where inflammation and tissue hypoxia are commonly induced (96).

Under hypoxic conditions in diseased tissues, HIF-1α activates glycolysis to generate ATP and induces proinflammatory cytokines when mitochondrial respiration is limited (35, 97). Numerous studies suggest that HIF-1α is strongly linked to the progression of atherosclerosis and metabolic disorders (57, 98). As prolonged hypoxia and inflammation become chronic, the pro-inflammatory state gradually shifts toward a lipid-metabolic adaptation as HIF-1α activity declining as HIF-2α progressively increases in a timely manner (13). This sequential activation of HIF isoforms represents a temporal and functional continuum linking the initiation of inflammation to its metabolic resolution.

HIF-2α mediates lipid metabolism and anti-inflammatory responses that promote tissue protection and recovery at the later adaptive phase of hypoxia. Although the HIF-2α-mediated regulation of lipid metabolism has not been as extensively studied as that of HIF-1α, the anti-inflammatory role of HIF-2α is possibly related to the protection or resolution of atherosclerosis. HIF-2α activates fatty acid β-oxidation to expend ATP and suppress inflammation in macrophages; thus, it is possible to ameliorate atherosclerosis (58, 98). Further evidence suggests that macrophage HIF-2α induces arginase 1 and downregulates molecules involved in the harmful effects of atherosclerosis, including NO and pro-inflammatory cytokines (14, 99). Arginase 1 is a representative anti-inflammatory marker regulated by HIF-2α in hypoxic conditions (14, 100). Arginase 1 promotes continuous efferocytosis, which is driven by the metabolism of arginine derived from apoptotic cells in the plaque, a process required for the regression of atherosclerosis (49). Although this clear evidence shows that arginase 1 is involved in resolving atherosclerosis, whether HIF-2α directly affects atherosclerosis remains unproven. However, HIF-2α-mediated regulation of ceramide biology offers mechanistic insight into how hypoxia and lipid metabolism converge to influence disease outcomes in tissues such as the liver, gut, and vasculature (17, 50, 85).

Nevertheless, several studies have reported that HIF-2α activation may exacerbate inflammation and metabolic dysregulation under certain pathological conditions. For example, HIF-2α activation in macrophages enhances IL-6 and TNF-α expression that sustains chronic inflammation in tumor and acute inflammatory models (51). Similarly, endothelial HIF-2α downregulates tissue factor pathway inhibitor (TFPI), thereby increasing pro-thrombotic and pro-inflammatory potential under hypoxic stress (101). These findings suggest that excessive or prolonged HIF-2α activation may contribute to maladaptive inflammatory or metabolic responses depending on the tissue context. This cross-tissue coordination underscores that hypoxia-responsive HIF signaling not only orchestrates macrophage metabolic programming but also integrates systemic immune-metabolic communication across organs.

Together, these mechanisms involving HIF-1α and HIF-2α suggest that HIF-1α predominantly sustains inflammatory glycolytic metabolism during acute hypoxia, whereas HIF-2α restores lipid and redox balance under chronic hypoxia, thereby supporting inflammation resolution and tissue stability. Collectively, the complex paradigms may suggest in this review indicate that the interplay between HIF-1α and HIF-2α defines a temporal and functional axis of macrophage metabolism integrating a unified mechanistic framework that links hypoxia, lipid metabolism, and immune regulation in atherosclerosis in a timely manner.

## 5 Therapeutic interventions using agonists and antagonists of HIFs

As previously emphasized, HIF signaling plays an important role in various diseases and suggests that the role of HIF may differ among cell types. Therefore, researchers are focusing on the development of HIF pathway–targeting agents. Table 1 provides a summary of the therapeutic pipeline involving HIF-targeting agonists and antagonists.

**Table 1:** Summary therapeutic agents targeting HIF pathways and their clinical status.

No. drug	Description	Clinical status	References
HIF-PHD inhibitors			
Daprodustat	PHD inhibitor that stimulates the production of endogenous erythropoietin (EPO) to treat anemia in patients with CKD.	FDA approved (2023, U.S.); also approved in EU and Japan	(111, 112)
Vadadustat	Increases EPO production via HIF stabilization; approved as a treatment for renal anemia in dialysis patients	FDA approved (2024)	(113)
Roxadustat	First-in-class HIF-PHI for anemia in CKD; \modulates iron metabolism and EPO.	Approved in China, Japan, South Korea; FDA denied due to safety concerns	(102)
Desidustat	Oral HIF-PHI approved in India as an alternative to ESA therapy in renal anemia.	India approved (2022); undergoing global expansion	(114, 115)
Enarodustat	Japanese-approved agent for renal anemia with selective PHD inhibition.	Approved in Japan (2020); other country trials ongoing	(116, 117)
HIF-2α inhibitors			
Belzutifan (PT2977)	Selective HIF-2α inhibitor that suppresses tumor growth in VHL-associated RCC and hypoxia-driven cancers.	FDA approved (2021) for VHL-related RCC and expanding in multiple tumor types	(108)
PT2385	First-in-class small molecule HIF-2α inhibitor; investigated in advanced RCC before Belzutifan development.	Completed Phase 1/2; development discontinued	(108)
Casdatifan (AB521)	Novel HIF-2α inhibitor under clinical evaluation for ccRCC and other solid tumors with hypoxic signatures.	Phase 1 trial active; Arcus-led ARC-20 study	(118)
DFF332	Orally bioavailable, selective HIF-2α inhibitor developed by Novartis targeting VHL disease and solid tumors.	Phase 1/2;	(119)
HIF-1α inhibitors			
PX-478	Selectively inhibits HIF-1α at transcription/translation levels; displays anticancer effects in solid tumors and lymphomas.	Phase 1 completed; further trials in planning	(106)
EZN-2968	An antisense oligonucleotide that targets HIF-1α mRNA can selectively inhibit HIF-1α at the transcriptional and translational levels. This treatment has demonstrated efficacy in patients with solid tumors.	Early clinical data available; development paused	(120, 121)

HIF, hypoxia-inducible factor; HIF-1α, hypoxia-inducible factor-1 alpha; HIF-2α, hypoxia-inducible factor-2 alpha; CKD, chronic kidney disease; RCC, renal cell carcinoma; ccRCC, clear cell renal cell carcinoma; PHD inhibitor; prolyl hydroxylase domain inhibitor; EPO, erythropoietin; HIF-PHI, hypoxia-inducible factor prolyl hydroxylase inhibitor; ESA, erythropoiesis-stimulating agent; VHL, von hippel-lindau; mRNA, messenger ribonucleic acid.

Among these, FG-4592 (Roxadustat) is the first HIF-PHD inhibitor approved in several countries for the treatment of patients with chronic kidney disease (CKD) (102). It stabilizes HIF signaling by inhibiting the oxygen-dependent degradation of the HIF-α subunit. The stabilization of HIF not only suggests potential treatments for metabolic disorders and inflammation in addition to anemia but has also been shown to be beneficial in a variety of diseases. FG-4592 has been shown to indirectly exert protective effects against atherosclerosis by promoting ceramide degradation through HIF stabilization in adipose tissue (17). It also regulates the NLRP3 inflammasome via HIF-2α in macrophages, as mentioned in the previous section, and simultaneously increases FAO-related gene expression while reducing insulin resistance (58). Thus, various metabolic effects have been reported for Roxadustat, including reducing total cholesterol and regulating iron metabolism and anti-inflammatory effects (103, 104). However, the non-selective HIF antagonists and agonists that underpin these benefits have simultaneously raised concerns about unexpected vascular and systemic side effects during long-term treatment. Indeed, clinical studies of HIF-PHD inhibitors such as Roxadustat have reported increased blood pressure and a higher incidence of adverse events with prolonged administration (105).

Agonists broadly activate HIFs, producing the beneficial effects mentioned above. Conversely, inhibition of HIF signaling is required in cancer therapy, where HIF activation promotes tumor progression and angiogenesis. In this setting, selective inhibition strategies targeting specific HIF isoforms are required to minimize adverse effects that impact chronic inflammatory diseases, metabolic disorders, and atherosclerosis due to non-selective HIF regulation. HIF specific inhibitors selectively block transcriptional activity by interfering with HIF-α translation, dimerization, or DNA binding. For example, PX478 is an HIF-1α inhibitor developed for the treatment of solid tumors and lymphoma that suppresses transcriptional activity by reducing HIF-1α protein levels. Although it was discontinued in phase 1 clinical trials, it has been tested in various preclinical and clinical studies (106). Since HIF-1α plays a key role in atherosclerosis, chronic mouse models have suggested its potential as an anti-atherogenic drug target (107). In murine models of atherosclerosis, treatment with a HIF-1α inhibitor has been shown to attenuate disease progression. Thus, HIF-1α inhibitors may have potential as anti-atherosclerotic agents. However, no clinically approved drugs currently exist that specifically inhibit HIF-1α, and this absence may allow inflammation within macrophages to persist or even worsen.

Next, HIF-2α selective inhibition has been developed as a therapeutic strategy for clear cell renal cell carcinoma (ccRCC), based on its ability to block HIF-2α–dependent transcription by preventing heterodimerization between HIF-2α and ARNT (108). The predecessor drug PT2385 demonstrated therapeutic efficacy in preclinical studies for multiple conditions, including pulmonary hypertension, and neurological disorders (109, 110). It was also shown to alleviate atherosclerosis in a mouse model by inhibiting the ceramide pathway mediated by intestinal HIF-2α activation induced by Candida albicans (89). However, subsequent HIF-2α inhibitors such as PT2977 have not been sufficiently studied in inflammatory or cardiovascular diseases outside of cancer. Since HIF-2α regulates both metabolic and inflammatory phenotypes in macrophages, it may exacerbate inflammatory diseases including atherosclerosis (17, 58). Therefore, further research is required to understand the effects of selective HIF-2α inhibitors or agonists in the context of atherosclerosis.

Taking together, HIF pathway may influence atherosclerotic plaque formation and progression by critically regulating macrophage lipid metabolism and inflammatory activity. However, given its deep involvement in broader immune–metabolic programs, caution is warranted with systemic or long-term modulation. Considering these limitations, strategies that precisely regulate macrophage-centric HIF signaling, rather than systemic approaches, may offer the potential to reprogram metabolic and inflammatory pathways associated with atherosclerotic progression.

## 6 Conclusion and future perspectives

Recent transcriptomic analysis have suggested that diverse subtypes of macrophages may perform distinct roles contributing to the progression or regression of atherosclerosis in the lesion area. Subsequent research has focused on identifying key regulators that determine various macrophage phenotypes in relation to atherosclerosis, employing three key terms: hypoxia, inflammation, and lipid metabolism. Among them, hypoxia is highlighted due to HIFs, which are known to modulate inflammatory responses and lipid metabolic pathways in macrophages. Notably, several studies have reported distinguished roles of HIF-1α and HIF-2α with similarities to the functional heterogeneity of lesional macrophage subtypes. Furthermore, the role of HIFs is actively investigated in atherosclerosis and other lipid-metabolism-related inflammatory diseases under hypoxic conditions. For example, HIF-1α drives proinflammatory functions by enhancing glycolysis, while also regulating lipid metabolism through genes such as *Lgals3*, *Cd36*, and *Plin2*. These findings have contributed to our current understanding of how hypoxia, inflammation, and lipid metabolism are intricately interconnected in the context of atherosclerosis. The activation of HIF-2α has been shown in some contexts to enhance lipid storage and anti-inflammatory properties in macrophages. Additionally, intestinal and hepatic HIF-2α can indirectly modulate the metabolism and inflammatory function of macrophages. These results suggest that HIF-2α may act as both a local and systemic regulator within the immuno-metabolic axis, providing a novel and effective therapeutic strategy for atherosclerosis rather than focusing solely on immune cells in the inflammatory plaque. Finally, it is increasingly recognized that atherosclerosis may represent a systemic immuno-metabolic disorder involving the intestine-liver-adipose tissue-immune axis. At this point, HIF signaling appears to play an important regulatory role in coordinating intercellular and systemic metabolic processes. Collectively, targeting HIF-signaling may offer a promising strategy to suppress the progression and potentially promote the regression of atherosclerosis.
